# High levels of heat stress among sugarcane workers in Thailand

**DOI:** 10.1093/annweh/wxaf002

**Published:** 2025-02-03

**Authors:** Tadpong Tantipanjaporn, Andrew Povey, Holly A Shiels, Martie van Tongeren

**Affiliations:** Centre for Occupational and Environmental Health, School of Health Sciences, Faculty of Biology, Medicine and Health, University of Manchester, Ellen Wilkinson Building (Block C), Oxford Road, Manchester, M13 9PL, United Kingdom; Division of Occupational Health and Safety, Faculty of Public Health, Naresuan University, 99 Moo 9, Thapo Sub-district, Muang District, Phitsanulok City, 65000, Thailand; Centre for Occupational and Environmental Health, School of Health Sciences, Faculty of Biology, Medicine and Health, University of Manchester, Ellen Wilkinson Building (Block C), Oxford Road, Manchester, M13 9PL, United Kingdom; Division of Cardiovascular Sciences, School of Medical Sciences, Faculty of Biology, Medicine and Health, University of Manchester, Core Technology Facility, 46 Grafton Street, Manchester, M13 9NT, United Kingdom; Centre for Occupational and Environmental Health, School of Health Sciences, Faculty of Biology, Medicine and Health, University of Manchester, Ellen Wilkinson Building (Block C), Oxford Road, Manchester, M13 9PL, United Kingdom

**Keywords:** climate change, heat symptoms, wet bulb globe temperature, WBGT, agricultural workers, harvest

## Abstract

**Objectives:**

With continued global warming, the effects of elevated temperatures on the health of agricultural workers are a particular concern. This study characterized the levels of heat stress in Thai sugarcane workers and investigated whether season and harvesting method were associated with it.

**Methods:**

Three hundred sugarcane workers in Nakhon Sawan Province, Thailand, were recruited, and information on demographics, working conditions, and clothing characteristics was collected from participants during the cooler months (*n* = 152 participants, mid-January to mid-February) and hotter month (*n* = 148, March). Heat stress was measured using the Wet Bulb Globe Temperature (WBGT) index, and the WBGT instruments were operated for a full work shift in the sugarcane fields where the participants worked. One-hour time weighted average (TWA) effective WBGT (WBGT_eff_-1hrTWA) estimates were determined for different times of the day based on the measured WBGT and clothing adjustment factor.

**Results:**

The average WBGT_eff_-1hrTWA in the cooler months ranged from 22.5 °C during the early morning to 31.3 °C during the hottest time of the day, and for the hotter month, it ranged from 25.4 °C to 33.9 °C, respectively. The measured WBGT, natural wet-bulb temperature (Tnwb), dry-bulb temperature (Tdb), globe temperature (Tg), air velocity (Av), and absolute water vapor pressure (e_a_) were all statistically significantly higher in the hotter month than in the cooler months. Harvesting during the hotter month and harvesting burnt sugarcane were significantly associated with increased effective WBGT. The harvesters’ heat stress in both seasons exceeded the American Conference of Governmental Industrial Hygienists - Threshold limit value for 72.7% of the working time in the cooler months and 90.9% in the hotter month.

**Conclusions:**

The heat stress in Thai sugarcane workers was high in both seasons, particularly in the hotter month and when harvesting burnt sugarcane. This results in a very high risk of developing heat-related health effects, and measures are needed to reduce heat stress. Heat stress in agricultural and other outdoor work in tropical climates is an immediate and growing problem.

**What’s Important About This Paper?**

This study found that Thai sugarcane workers frequently experienced levels of heat stress during both the “cool” and “hot” harvesting periods that exceeded established standards. Collective action to develop interventions that mitigate heat stress exposure among the sugarcane workforce is urgently needed.

## Introduction

Agricultural workers in tropical countries can be exposed to extreme heat and are therefore at increased risk of heat-related illnesses (Occupational Safety and Health Administration, 2022), accidents, and injuries (Spector et al. 2016; Wasterlund 2018), decreasing labor productivity (Lundgren et al. 2014; Radir *et al.*, 2017; Wasterlund 2018; Sadiq et al. 2019). Agriculture has been a major sector in the economic development of Thailand (Statistic Research Department, 2023). In 2023, sugarcane was the leading crop in Thailand, with approximately 105.9 million metric tons produced by approximately 100,000 (mostly) small farms (National Statistical Office, 2023).

Thailand is located in the tropics, so the country is generally hot and humid, particularly from March through June (Kjellström et al. 2019; World Population Review 2024). Thailand has 3 seasons: the rainy season (mid-May to mid-October), the cool season (mid-October to mid-February), and the hot season (mid-February to mid-May) (Thawillarp et al. 2015). Thailand’s average temperature in 2023 was 28.1 °C, which was the highest average annual temperature in over 73 yr (1951 to 2023), and the peak was 44.6 °C, occurring in April (Thai Meteorological Department 2023b). The average annual temperature in Thailand has increased by 0.7 °C, over the last century (Kjellström et al. 2019; World Population Review 2024).

Heat stress in occupational settings refers to total body heat load, which is a combination of metabolic heat, environmental factors (eg air temperature, humidity, air movement, and radiant heat), and clothing (American Conference of Governmental Industrial Hygienists 2022). The wet bulb globe temperature (WBGT) index is a measure of heat stress that represents the thermal environment to which an individual is exposed (International Organization for Standardization 2017). Heat stress exposure levels can be compared with the exposure limit for heat stress, for example, the health-based limit set by the American Conference of Governmental Industrial Hygienists (ACGIH). There are 2 heat stress limits set by ACGIH: 1 threshold limit value (TLV) and 1 action level (AL). The purpose of the TLV is to maintain core body temperature below 38 °C, and it indicates conditions believed to allow almost all heat-acclimatized, adequately hydrated, unmedicated, healthy workers to be repeatedly exposed to heat without negative health consequences. The AL is similarly protective of unacclimatized workers and suggests conditions that require consideration in order to properly manage heat stress. The ACGIH guidelines recommend that the effect of clothing on heat loss should be considered and that if a worker wears heavier clothing, the exposure limit should be lowered (American Conference of Governmental Industrial Hygienists 2022). Few studies in the agricultural sector have considered this issue in assessing and interpreting heat stress. Only a single study by Wagoner et al. (2020) interpreted heat stress in terms of effective WBGT adjusted dependent on clothing.

Sugarcane farming has 2 main labor-intensive stages: planting and harvesting. In the sugarcane harvesting stage, there are generally 2 main methods: harvesting unburnt cane and harvesting burnt cane (Chaya 2024). For burnt sugarcane harvesting, the sugarcane is burnt the day before manual harvesting to reduce the amount of straw (sugarcane leaves and other plant material) thereby making the fields cleaner and easier to harvest. Most of the leaves are burnt off, but the stalk remains unburnt. When harvesting unburnt sugarcane, there are 2 main steps. First the leaves need to be removed, then the stalk needs to be cut down (Pongpat et al. 2017; Boonruksa et al. 2020; Moitinho et al. 2021). The harvesting method could affect heat stress. For example, leaves remaining on unburnt sugarcane might provide a small amount of shade.

Sugarcane harvesters in Cambodia (Radir *et al.*, 2017) and Costa Rica (Crowe et al. 2011, 2013) perform a heavy workload and are exposed to high levels of WBGT, which exceed standards established by ACGIH and the Occupational Safety and Health Administration (OSHA). Although a few studies in Thailand have reported that heat exposure in sugarcane fields exceeds safety standards for heat stress (Boonruksa et al. 2020; Pundee et al. 2021; Wichatorn and Chaiklieng 2022), research on heat stress in sugarcane fields in Thailand are still very limited. Sugarcane workers work outdoors in fields where they generally perform heavy physical labor and are exposed to extreme heat for approximately 8 to 11 h a day. The problem of mitigating heat stress in these workers is compounded by the informal nature of the sugarcane workforce; Thai regulatory standards of occupational health and safety regarding heat stress apply only to formal workers who are officially recorded by the government (Boonruksa et al. 2020). The purpose of this study was to characterize heat stress levels of Thai sugarcane workers across the harvest season in the cooler months and in the hot months. Another objective was to determine whether heat stress varies with season and harvesting method.

## Materials and methods

### Study design, setting, time, and participants

This was a cross-sectional study undertaken in Khao Kala Subdistrict in the Phayuha Khiri District of Nakhon Sawan Province in northern Thailand, a province with one of the largest sugarcane growing areas in Thailand (Office of the Cane and Sugar Board 2022). The subdistrict and district were chosen randomly from all subdistricts and districts in the province. Sugarcane worker camps (locations where they live during harvesting period) in Khao Kala Sub-district were selected using convenience sampling. Sugarcane workers from a total of 13 camps participated. A single crop of sugarcane is grown annually in Thailand, and the harvest season ranges from December to March (Boonruksa et al. 2020). The study was carried out for 14 d in the harvesting stage during the cooler months (mid-January to mid-February 2023) and 11 d in the hot month (March 2023), with the agreement of farmers (sugarcane field owners) and participants. The participants in this study were 300 individual sugarcane workers harvesting sugarcane. There were 152 participants during the cooler months and 148 in the hot month. All participants in this study were at least 18 yr of age and able to read, write, and communicate in Thai. The study protocol was reviewed and approved by the University Research Ethics Committee of The University of Manchester (Ref No. 2022-15332-26084), and also by the Institutional Review Board of Naresuan University, Thailand (IRB No. P2-0368/2565). All the participants were fully informed of the nature of the study in advance, and each provided a signed consent form agreeing to participate in the study.

### Heat stress measurements and workload assessments

To assess heat stress exposure, 2 WBGT instruments (Brand: TSI Quest, Model: QUESTemp 34, Calibrated date: 9 January 2023) were placed in each field, according to ISO 7243:2017 (International Organization for Standardization 2017). The WBGT is a measure of the heat stress in direct sunlight, which takes into account: temperature, humidity, wind speed, sun angle, and cloud cover (American Conference of Governmental Industrial Hygienists 2022). Exact positioning of the 2 instruments varied depending on the particular field and the position of the participants. They were basically placed at locations close to where the participants were cutting (ie both at the cutting edge), with one instrument placed towards the middle of the field and the other near one of the field’s borders. The instruments were moved several times a day, following the harvesting line to remain close to where the participants were cutting the sugarcane. Some participants needed to work in more than one field in a single day. A common reason was that sometimes harvesting was completed in one field, so the participants moved to a new field. When this happened, instruments were moved to the new field and positioned as before. In the new field, the participants might harvest sugarcane with a different method (burnt or unburnt), and this was determined by the sugarcane field owners. The instruments were operated for a full work shift (approximately 06:00 to 17:00). The instruments were calibrated daily using the verification module both before and after measurements. The instruments were set at the chest level of the participants, and the sensors were allowed to stabilize for 15 min before pressing the Run/Stop key to log the day’s data. The instruments logged the data every 5 min throughout the work shift and provided: natural wet-bulb temperature (*Tnwb*), dry-bulb temperature (*Tdb*), globe temperature (*Tg*), relative humidity (*Rh*), and measured WBGT Outdoor. The measured WBGT Outdoor was calculated based on Equation (1) (American Conference of Governmental Industrial Hygienists 2022).


$$\begin{array}{lll} & Measured\;\;\;WBGT\;\;\;(outdoor)\; & =0.7\;\;\;Tnwb+0.2\;\;\;Tg+0.1\;\;\;Tdb \end{array}$$ (1)

In the case of working in more than one field in a single day, a TWA of the WBGT was calculated to accurately include and reflect the time spent in each location, based on Equation (2).


$$\begin{array}{llll} & Average\;measured\;WBGT\; & =(WBGT_{1}\times T_{1}+WBGT_{2}\times T_{2}\; & +\ldots +WBGT_{n}\times T_{n})/(T_{1}+T_{2}+\ldots +\;T_{n}) \end{array}$$ (2)

where *WBGT*_1_, *WBGT*_2_, and *WBGT*_*n*_ represent the measured WBGT for each work location, and *T*_1_, *T*_2_, and *T*_*n*_ represent the time spent in each work location.

The measured WBGT results from the 2 instruments were averaged and then used to calculate the effective WBGT as shown in Equation (3), because WBGT alone represents only the environment, it needs to be adjusted for the effect of clothing. According to the ACGIH-clothing adjustment factor (CAF) guidelines, where a standard cotton shirt and trousers is 0 °C, the ACGIH-CAF for “double layer cloth (woven) clothing” is 3 °C (American Conference of Governmental Industrial Hygienists 2022). Those participants wearing 2 layers of standard shirts had the ACGIH-CAFs of 3 °C added to the measured WBGT, whereas for the participants with one layer the measured WBGT was used. For those participants wearing 3 layers, the ACGIH-CAF for double-layer clothing (3 °C) was also applied because there is no CAF guideline for more than 2 layers of clothes.


$$\begin{array}{lll} Effective\;\;\;WBGT= & \;the\;\;\;average\;\;\;measured\;\;\;WBGT\; & +Clothing\;\;\;Adjustment\;\;\;Factor \end{array}$$ (3)

The WBGT index and the metabolic work rates (workload) are together generally considered to determine the potential for heat stress among people exposed to hot environments. In this study, the metabolic rate of the sugarcane harvesters was determined using ACGIH guidelines for workload categories, specifically “light” (metabolic rate = 180 W), “moderate” (300 W), “heavy” (415 W), or “very heavy” (520 W). The guidelines provide example activities for each of these categories. The approximate proportion or percentage of work and breaks within an hour was calculated to select the WBGT criterion for the TLV and AL. To determine the percentage of work and recovery (breaks) within the total cycle, the work or break times were divided by the total cycle time and multiplied by 100 (American Conference of Governmental Industrial Hygienists 2022).

To compare this study’s results with the standards for heat stress, a 1-h TWA effective WBGT (WBGT_eff_-1hrTWA) was calculated. This follows the guidelines that the WBGT_eff_-1hrTWA, which averages the results over a 1-h period, is used for continuous multiple-hour or all-day exposures (International Organization for Standardization 2017; Occupational Safety and Health Administration 2017). The WBGT_eff_-1hrTWA was calculated throughout the full work shift and compared with the ACGIH-TLV and AL for heat stress based on the intensity of the workload and the allocation of work in a cycle of work and recovery. Based on metabolic rate categories, WBGT criteria for TLV and AL can be calculated using Equations (4) and (5), respectively (American Conference of Governmental Industrial Hygienists 2022).


$$\begin{array}{l} TLV=56.7\;11.5^{*}log10\;M \end{array}$$ (4)

$$\begin{array}{l} AL=59.9\;14.1{}^{*}log10\;M \end{array}$$ (5)

where *M* represents metabolic rate in watts (W).

### Ambient air velocity (*Av*) measurement

Ambient air velocity (*Av*) was assessed using an air velocity meter (Brand: TSI, Model 9515, Calibrated date: 9 November 2022). It was measured at 6 times each day, spanning both morning (8:00, 10:00, and 12:00) and afternoon (13:00, 15:00, and 17:00) at the same 2 fixed points used for measuring WBGT. Each measurement was taken 3 times with a 1-min interval, and the average value was calculated.

### Absolute water vapor pressure (*e*_*a*_)

Absolute water vapor pressure (*e*_*a*_), also known as vapor pressure, is an indirect indicator of the water vapor content in the atmosphere (Qiu et al. 2021). The *e*_*a*_ was calculated from the measured Rh and saturation vapor pressure (*e*_*s*_) using Equation (6) (Wei et al. 2021), while the *e*_*s*_ was calculated from the measured *Tdb* using Equation (7) (National Weather Service, 2024).


$$\begin{array}{l} e_{a}=(Rh*e_{s})/100 \end{array}$$ (6)

$$\begin{array}{l} e_{s}=6.11^{*}10(7.5^{*}Tdb)/(237.3^{*}Tdb)) \end{array}$$ (7)

### Questionnaire

The participants were interviewed by a research team of Field Representatives (FRs) who were trained and supervised by the Lead Researcher (LR, T. Tantipanjaporn). The FRs were trained by the LR to understand the questionnaire in detail. The questions included demographic characteristics, working conditions, and clothing characteristics (Supplementary Material). Since the participants were Thai, the process of translating (from English into Thai) and validating the questionnaire was conducted as follows: forward translation, synthesis of the translations, backward translation, review of the forward-backward translation, and validity of the questionnaire by 7 experts.

### Data analysis

Data were analyzed by SPSS software (version 29.0). Descriptive statistics were used to characterize the data. Categorical data were presented as frequency and percentage, while continuous data were presented as mean, standard deviation (SD), and minimum and maximum values. A univariate general linear model (GLM) was performed to test whether each independent variable (season and harvesting method) was associated with each heat stress parameter (measured *WBGT*, *Tnwb*, *Tdb*, *Tg*, *Rh*, *Av*, and *e*_*a*_). The measurement location (middle or edge) was included as a covariate in the analyses for adjustment. A multivariable GLM then analyzed all independent variables together with each heat stress parameter. Analyses were based on 50 stationary WBGT measurements (25 d, 2 locations per day) and 290 air velocity measurements (5 to 6 per day at each WBGT location). Subsequently, univariate and multivariable GLMs were conducted to assess whether season and harvesting method were associated with effective WBGT. These analyses were based on 300 individual effective WBGT results. Unfortunately, the starting time and ending time of the participants’ workday differed between the 2 seasons. For this reason, the analysis for both GLMs was run by including only data from the main portion of the workday, when there were parallel measurement times (08:30 to 16:00) for both seasons. *R*^2^ and adjusted *R*^2^ from GLMs were provided. A chi-square test and an independent *t*-test were used to analyze differences in general characteristics by seasons and shirt layers by harvesting methods.

## Results

### General characteristics

Table 1 shows general characteristics of the study participants. The results show that males outnumbered females among the participants (55.7% male and 44.0% female). Their average age was around 41. Their most common primary responsibility was “sugarcane harvesting only” (93.7%). Most participants wore 2 layers of shirts in the past 7 d (73.0%), and this was consistent in both the cooler and hotter months. A majority of the participants (81.3%) did not unbutton, unzip, or remove any layer of their clothing whenever they felt hot in the past 7 d. The method that the majority of participants (75.9%) used to try to keep cool during breaks was going into the shade of sugarcane plants or nearby trees. Others (19.7%) used man-made shade to provide temporary shade in the fields. Only “unbuttoned, unzipped, or removed clothing” and “methods to stay cool during breaks” differed significantly between the 2 seasons.

**Table 1.** General characteristics of the study participants (*n* = 300).

General characteristic	Total (*n* = 300)	Cooler months (*n* = 152)	Hotter month (*n* = 148)	*P*-value
Gender; *n* (%)				
Male	167 (55.7)	79 (52.0)	88 (59.5)	0.17^d^
Female	132 (44.0)	73 (48.0)	59 (39.9)	
Preferred not to say	1 (0.3)	0 (0)	1 (0.6)	
Age				
Mean ± SD	41.3 ± 11.6	40.7 ± 11.1	42.0 ± 12.2	0.32^e^
Range	18.0–81.0	20.0–65.7	18.0–81.0	
Main responsibility during the past 7 d of this sugarcane harvest; *n* (%)				
Sugarcane harvesting only	281 (93.7)	139 (91.4)	142 (95.9)	0.11^d^
Sugarcane harvesting plus one other task^a^	19 (6.3)	13 (8.6)	6 (4.1)	
Number of layers of shirt worn at work in the past 7 d^b^; *n* (%)				
One	67 (22.3)	31 (20.4)	36 (24.3)	0.46^d^
Two	219 (73.0)	112 (73.7)	107 (72.3)	
Three	14 (4.7)	9 (5.9)	5 (3.4)	
Unbuttoned, unzipped, or removed clothing when hot in the past week; *n* (%)				
No	244 (81.3)	116 (76.3)	128 (86.5)	0.02^d,f^
Yes	56 (18.7)	36 (23.7)	20 (13.5)	
Methods to help keep cool during breaks^c^; *n* (%)				
Natural shade: Stay under sugarcane or trees	277 (75.9)	144 (86.7)	133 (66.8)	<0.001^d,f^
Man-made shade: Use umbrella, cloth, or tent	72 (19.7)	20 (12.0)	52 (26.1)	
Water: Wash your face or use a pond	15 (4.1)	1 (0.6)	14 (7.0)	
Fans: Use fans for airflow	1 (0.3)	1 (0.6)	0 (0)	

^a^With counting sugarcane bundles (*n* = 5), with driving the leaf cutting vehicle (*n* = 1), with leveling sugarcane on a truck (*n* = 5), or with driving a tractor (*n* = 8).

^b^Types of shirt: T-shirt, shirt, or jacket made of polyester, cotton/polyester blend, or denim.

^c^Participants could select more than one: total *n* = 365, *n* = 166 for cooler months, and *n* = 199 for hotter month.

^d^Chi-square test; ^e^independent t-test; ^f^*P*-value < 0.05.

### Sugarcane harvesting methods

**Fig. 1.**
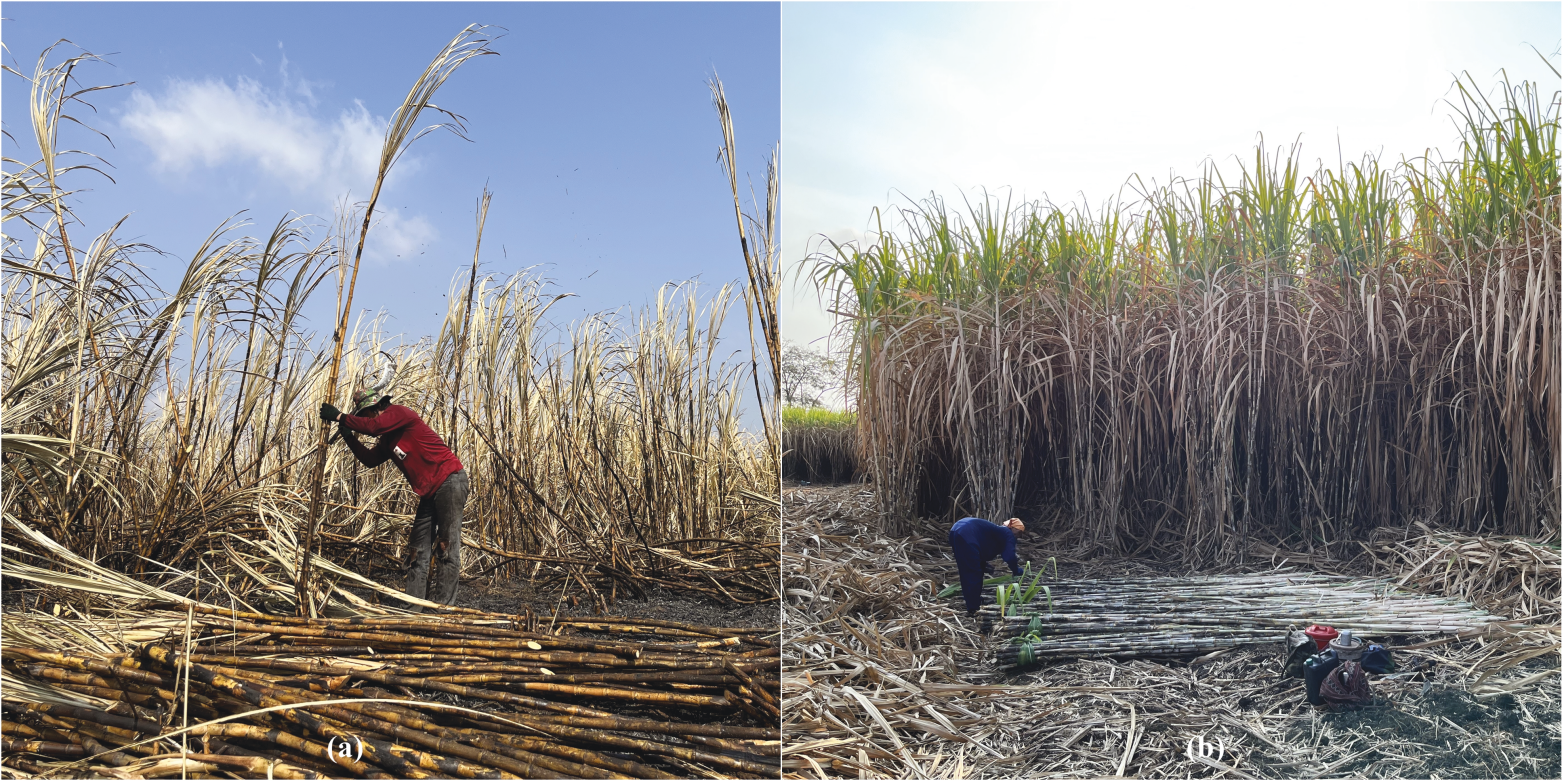
Harvesting a) burnt sugarcane and b) unburnt sugarcane.

There are 2 main methods for harvesting sugarcane: with burning (Fig. 1a) or without burning (Fig. 1b). Among the 14 d of data collection in the cooler months, harvesting of the sugarcane was most often performed without prior burning, observed on 57.2% of the data collection days during this season. Harvesting burnt sugarcane and harvesting burnt and unburnt sugarcane in 1 d during the cooler months were each observed on 21.4% of the data collection days. During the 11 d of the hotter month’s data collection, the sugarcane was most often burned prior to harvesting, happening on 54.5% of the days, followed by harvesting unburnt sugarcane (27.3%), and harvesting burnt and unburnt sugarcane in 1 d (18.2%).

### Number of shirt layers based on sugarcane harvesting methods

The number of participants wearing 1, 2, or 3 shirt layers did not differ between the 3 harvesting methods (*P*-value = 0.70). While harvesting burnt sugarcane, unburnt sugarcane, and both types in 1 d, the majority of the participants (75.4%, 73.1%, and 68.9%, respectively) wore 2 layers of shirt, followed by wearing 1 layer (18.9%, 23.1%, and 27.0%, respectively) and wearing 3 layers (5.7%, 3.8%, and 4.1%, respectively).

### Heat stress assessment

**Fig. 2.**
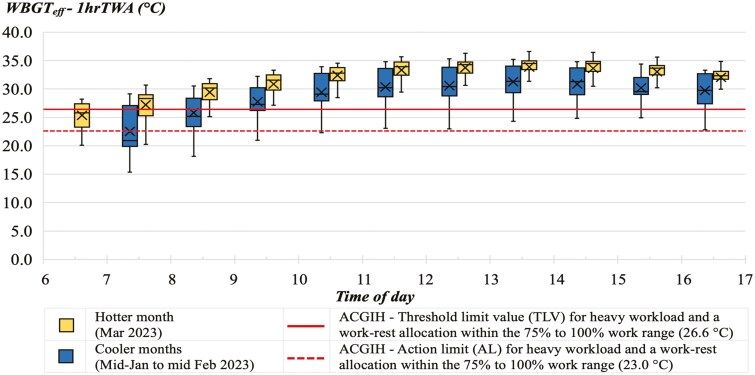
Hourly average 1-h TWA effective WBGT (WBGT_eff_-1hrTWA) throughout the working day during the cooler months (*n* = 152 participants) and hotter month (*n* = 148), A box and whisker plot illustrate the distribution of the data by presenting the 5-number summary, which includes the minimum score, first (lower) quartile, median, third (upper) quartile, and maximum score. Additionally, crosses within each box represent the mean value.

The harvesting tasks of the participants were observed to consist of manually cutting the sugarcane using a machete, stripping the leaves off, and piling stalks in rows on the ground. When the observed activities of the participants were compared with the example activities in the ACGIH guidelines, the participants’ workload fell into the “heavy workload” category corresponding to a representative metabolic rate of 415 W. The Thai sugarcane workers in this study had no official breaks or break rules. Their breaks were self-determined, typically involving informal pauses or breaks throughout the workday (eg stepping away for a few minutes). The results showed that the average working time was 10.1 h per day, and the average break time, including morning, lunch, and afternoon breaks, was 1.4 h. This gave a total cycle time (work and rest) of 11.5 h of which 87.8% were for work and 12.2% for recovery. This means that, with each hour, approximately 52.7 min (87.8%) were spent working and 7.3 min (12.2%) were spent on breaks. Therefore, the allocation of work and recovery time in this study fell within the 75% to 100% work range per hour, as outlined in the guidelines. Figure 2 shows hourly average WBGT_eff_-1hrTWA throughout the working day during the cooler and hotter month(s), compared with TLV and AL for heat stress, both based on heavy workload and a work-rest allocation within the 75% to 100% work range. The average WBGT_eff_-1hrTWA in the cooler months ranged from 22.5 °C during the early morning to 31.3 °C during the hottest time of the day, and for the hotter month, it ranged from 25.4 °C to 33.9 °C, respectively. The highest observed WBGT_eff_-1hrTWA in the cooler and hotter month(s) was 13:00 to 14:00, with the average values of 31.3 °C and 33.9 °C, with the highest levels observed at 35.2 °C and 36.6 °C, respectively. After comparing the WBGT_eff_-1hrTWA with the ACGIH-TLV at 26.6 °C, it was determined that the sugarcane harvesters were at high risk of heat stress for both seasons. In the cooler months, heat stress exposure of the harvesters exceeded the ACGIH-TLV for 72.7% of their working time (9:00 to 17:00). In the hotter month, heat stress exposure exceeded the ACGIH-TLV for 90.9% of their working time (7:00 to 17:00).

### Predicting heat stress parameters based on season and sugarcane harvesting method

The hotter month and burnt sugarcane harvesting had higher heat stress parameters, namely measured *WBGT*, *Tnwb*, *Tdb*, *Tg*, *Rh*, *Av*, and *e*_*a*_ (Table 2). Table 3 illustrates both univariate and multivariable analyses comparing heat stress parameters between seasons and harvesting methods. The univariate analyses revealed that measured *WBGT*, *Tnwb*, *Tdb*, *Tg*, *Rh*, *Av*, and *e*_*a*_ were statistically significantly higher in the hotter month than in the cooler months, and the multivariable analyses results were very similar. The univariate analyses showed that the measured *WBGT*, *Tnwb*, *Tdb*, *Rh*, and *e*_*a*_ were statistically significantly higher when harvesting burnt sugarcane compared to harvesting unburned sugarcane. However, in the multivariable analyses, only the differences in *Rh* and *e*_*a*_ remained significant. There were no statistically significant differences in *Tg* and *Av* between any of the harvesting methods. Table 4 shows univariate and multivariable analyses for the variables associated with individual heat stress exposure, in terms of effective WBGT. Multivariable analysis shows that the effective WBGT in the hotter month was significantly higher than the results in the cooler months [B (95% CI): 2.3 (1.7, 2.9)]. Effective WBGT among participants who harvested burnt sugarcane [B (95% CI): 1.7 (1.1, 2.4)] and those who did mixed harvesting in 1 d [B (95% CI): 1.1 (0.4, 1.8)] were significantly higher than those who harvested unburnt sugarcane.

**Table 2.** Results of heat stress parameters, separated by season and harvesting method.

	Measured WBGT (°C)	*Tnwb* (°C)	*Tdb* (°C)	*Tg* (°C)	*Rh* (%)	*Av* (m/s)	*e*_*a*_ (hPa)
**Overall results of heat stress parameters**							
	(*n* = 50)	(*n* = 50)	(*n* = 50)	(*n* = 50)	(*n* = 50)	(*n* = 290)	(*n* = 50)
Mean ± SD	28.6 ± 2.7	24.2 ± 3.0	31.8 ± 2.3	42.6 ± 3.1	33.5 ± 7.9	1.6 ± 1.0	16.1 ± 4.8
Range	22.7–32.9	18.1–27.8	26.7–36.1	35.6–49.6	17.7–50.7	0.04–5.1	7.0–22.5
**Heat stress parameters separated by seasons**							
* Cooler months*							
	(*n* = 28)	(*n* = 28)	(*n* = 28)	(*n* = 28)	(*n* = 28)	(*n* = 160)	(*n* = 28)
Mean ± SD	27.3 ± 2.6	22.7 ± 3.0	30.5 ± 1.9	41.7 ± 2.5	31.4 ± 8.5	1.5 ± 0.9	14.0 ± 4.8
Range	22.7–31.0	18.1–26.8	26.7–33.9	36.8–46.1	17.7–50.7	0.04–3.8	7.0–22.4
* Hotter month*							
	(*n* = 22)	(*n* = 22)	(*n* = 22)	(*n* = 22)	(*n* = 22)	(*n* = 130)	(*n* = 22)
Mean ± SD	30.4 ± 1.7	26.1 ± 1.5	33.6 ± 1.4	43.7 ± 3.4	36.0 ± 6.5	1.9 ± 1.0	18.7 ± 3.3
Range	25.5–32.9	21.9–27.8	30.8–36.1	35.6–49.6	23.5–45.2	0.2–5.1	11.6–22.5
**Heat stress parameters separated by harvesting methods**							
* Harvesting burnt sugarcane*							
	(*n* = 18)	(*n* = 18)	(*n* = 18)	(*n* = 18)	(*n* = 18)	(*n* = 100)	(*n* = 18)
Mean ± SD	29.7 ± 2.5	25.6 ± 2.7	32.7 ± 2.4	42.9 ± 3.0	37.0 ± 7.6	1.8 ± 1.0	18.5 ± 4.4
Range	24.9–32.9	19.9–27.8	28.8–36.1	37.8–49.0	22.6–50.7	0.2–4.4	9.4–22.5
* Harvesting unburnt sugarcane*							
	(*n* = 20)	(*n* = 20)	(*n* = 20)	(*n* = 20)	(*n* = 20)	(*n* = 118)	(*n* = 20)
Mean ± SD	27.5 ± 2.6	22.7 ± 3.0	30.8 ± 2.1	42.6 ± 2.3	29.3 ± 7.9	1.5 ± 0.8	13.3 ± 4.9
Range	22.7–31.0	18.1–26.8	26.7–33.9	36.8–46.1	17.7–42.9	0.04–4.1	7.0–21.6
* Mixed harvesting in 1 d* ^a^							
	(*n* = 12)	(*n* = 12)	(*n* = 12)	(*n* = 12)	(*n* = 12)	(*n* = 72)	(*n* = 12)
Mean ± SD	28.9 ± 2.5	24.7 ± 2.3	32.3 ± 1.8	42.0 ± 4.2	35.1 ± 5.3	1.6 ± 1.1	17.0 ± 2.2
Range	25.3–31.2	20.8–26.9	29.5–35.5	35.6–49.6	23.5–41.5	0.2–5.1	13.3–20.6

^a^Harvesting both burnt and unburnt sugarcane in 1 d.

Measured *WBGT* = Measured wet bulb globe temperature; *Tnwb* = natural wet-bulb temperature; *Tdb* = dry-bulb temperature; *Tg* = globe temperature; *Rh* = relative humidity; *Av* = air velocity; *e*_*a*_ = absolute water vapor pressure; *n* = 50 measurements (2 locations*25 d) for *WBGT*, *Tnwb*, *Tdb*, *Tg*, and *Rh*; *n* = 290 measurements (5 to 6 times*2 points*25 d) for *Av*; *e*_*a*_, calculated from *Rh* and *Tdb*, resulted in 50 values.

**Table 3.** Univariate and multivariable analyses comparing heat stress parameters between seasons and harvesting methods.

Explanatory variable	Measured WBGT		Tnwb		Tdb		Tg		Rh		Av		*e*_*a*_	
	B (95% CI)		B (95% CI)		B (95% CI)		B (95% CI)		B (95% CI)		B (95% CI)		B (95% CI)	
	UV^a^	MV^b^	UV^a^	MV^b^	UV^a^	MV^b^	UV^a^	MV^b^	UV^a^	MV^b^	UV^a^	MV^b^	UV^a^	MV^b^
**Seasons (Reference = Cooler months)**														
Hotter month	3.1^d^ (1.8, 4.3)	2.7^d^ (1.3, 4.1)	3.4^d^ (2.0, 4.8)	2.8^d^ (1.2, 4.3)	3.1^d^ (2.1, 4.0)	2.9^d^ (1.8, 3.9)	2.0^c^ (0.3, 3.6)	2.3^c^ (0.4, 4.2)	4.6^c^ (0.2, 9.0)	2.0 (-2.5, 6.7)	0.4^d^ (0.2, 0.7)	0.4^d^ (0.2, 0.7)	4.7^d^ (2.3, 7.0)	3.3^c^ (0.7, 5.9)
**Harvesting methods (Reference = Harvesting unburnt sugarcane)**														
Burnt sugarcane	2.3^c^ (0.6, 3.9)	1.0 (−0.6, 2.6)	2.9^c^ (1.1, 4.6)	1.6 (−0.2, 3.3)	1.9^c^ (0.5, 3.3)	0.6 (−0.6, 1.8)	0.4 (−1.7, 2.4)	−0.7 (−2.8, 1.4)	7.7^c^ (3.0, 12.5)	6.8^c^ (1.9, 11.6)	0.2 (−0.1, 0.5)	−0.01 (−0.3, 0.3)	5.2^d^ (2.4, 8.0)	3.6^c^ (0.6, 6.5)
Mixed harvesting^e^	1.4 (−0.4, 3.3)	0.6 (−1.1, 2.3)	2.0 (−0.02, 4.0)	1.2 (−0.7, 3.0)	1.6 (−0.01, 3.1)	0.7 (−0.6, 2.0)	−0.6 (−2.8, 1.7)	−1.3 (−3.5, 1.0)	5.8^c^ (0.5, 11.1)	5.2 (−0.4, 10.8)	0.1 (−0.2, 0.4)	−0.04 (−0.3, 0.2)	3.7^c^ (0.6, 6.9)	2.7 (−0.4, 5.8)
**Measurement locations (Reference = Edge)**														
Middle	−0.03 (−1.6, 1.5)	−0.03 (−1.3, 1.3)	−0.01 (−1.7, 1.7)	−0.01 (−1.4, 1.4)	−0.05 (−1.3, 1.3)	−0.05 (−1.0, 0.9)	−0.08 (−1.8, 1.7)	−0.08 (−1.7, 1.6)	0.2 (−4.1, 4.8)	0.2 (−3.9, 4.4)	0.01 (−0.2, 0.2)	0.01 (−0.2, 0.2)	0.01 (−2.8, 2.8)	0.01 (−2.3, 2.3)
*R* ^2^ for MV	N/A	0.35	N/A	0.37	N/A	0.49	N/A	0.13	N/A	0.21	N/A	0.05	N/A	0.34
Adjusted *R*^2^ for MV	N/A	0.29	N/A	0.32	N/A	0.44	N/A	0.05	N/A	0.14	N/A	0.04	N/A	0.28

^a^Univariate General Linear Model; ^b^Multivariable General Linear Model; ^c^*P*-value < 0.05, ^d^*P*-value < 0.001.

^e^Harvesting both burnt and unburnt sugarcane in 1 d.

There were only these 3 variables in multivariable analysis.

*R* ^2^ from univariate general linear models was provided in Supplementary Table S1.

Measured *WBGT* = Measured wet bulb globe temperature; *Tnwb* = natural wet-bulb temperature; *Tdb* = dry-bulb temperature; *Tg* = globe temperature; *Rh* = relative humidity; *Av* = air velocity; *e*_*a*_ = absolute water vapor pressure; *n* = 50 measurements (2 locations*25 d) for *WBGT*, *Tnwb*, *Tdb*, *Tg*, and *Rh*; *n* = 290 measurements (5 to 6 times*2 points*25 d) for Av; *e*_*a*_, calculated from *Rh* and *Tdb*, resulted in 50 values.

**Table 4.** Univariate and multivariable analyses for the variables associated with individual heat stress (effective WBGT) (*n* = 300).

Explanatory variable	Univariate analysis^a^	Multivariable analysis^a^
	B (95% CI)	B (95% CI)
**Seasons (Reference = Cooler months)**		
Hotter month	3.0 (2.4–3.5)^c^	2.3 (1.7–2.9)^c^
**Harvesting methods (Reference = Harvesting unburnt sugarcane)**		
Burnt sugarcane	2.9 (2.3–3.6)^c^	1.7 (1.1–2.4)^c^
Mixed harvesting^d^	1.7 (1.0–2.5)^c^	1.1 (0.4–1.8)^b^

Univariate analysis *R*^2^: 0.28 (season), 0.21 (harvesting method). Multivariable analysis *R*^2^: 0.34, adjusted *R*^2^: 0.33.

^a^General Linear Models, ^b^*P*-value < 0.05, ^c^*P*-value < 0.001.

^d^Harvesting burnt and unburnt sugarcane in 1 d.

There were only these 2 variables in multivariable analysis.

## Discussion

This study assessed heat stress in Thai sugarcane workers during the cooler and hotter month(s) of the harvest season and compared the results with established heat stress standards. The environmental factors related to heat stress were also determined. Heat stress measured using WBGT was high in both seasons, and slightly higher in the hotter month. The participants’ heat stress was above the levels set by ACGIH as safe work conditions. With the exception of Rh, all the components that contribute to WBGT were significantly higher in the hotter month. Effective WBGT was higher when harvesting burnt sugarcane.

It is difficult to compare the heat stress results from this current study to previous studies, because none of the previous studies adjusted WBGT results for CAF, and they also had different methods for measuring time (Boonruksa et al. 2020; Pundee et al. 2021; Wichatorn and Chaiklieng 2022). Research on heat stress measured as effective WBGT in sugarcane or other agricultural workers is still limited. Only a single study of grape farmworkers in Mexico adjusted WBGT for clothing (Wagoner et al. 2020). That study, like the current one, found that WBGT results exceeded the ACGIH-TLV. Pundee et al. (2021) studied Thai sugarcane harvesters and found that the average TWA-WBGT result in sugarcane harvesting fields was 27.9 °C with a range of 27.0 °C to 29.8 °C, measured in the hot season. Boonruksa et al. (2020), who also studied heat stress among Thai sugarcane harvesters, reported that the average TWA-WBGT result was 30.6 ± 2.0 °C, with a range of 24.1 °C to 33.9 °C, measured in the hot season. Wichatorn and Chaiklieng (2022) studied Thai sugarcane harvesters and found that average WBGT results in the cold and hot seasons were 30.2 °C and 31.2 °C, respectively. The results in this study are consistent with the high results observed in previous studies (Boonruksa et al. 2020; Pundee et al. 2021; Wichatorn and Chaiklieng 2022). However, the results in this study were even higher than what has been previously reported. This study found that harvesting workload was heavy, with a work-rest allocation of 87.8% following the ACGIH guidelines. Previous researchers in Thailand (Boonruksa et al. 2020; Pundee et al. 2021; Wichatorn and Chaiklieng 2022), Cambodia (Radir *et al.*, 2017), and Costa Rica (Crowe et al. 2013) also assessed the workload of sugarcane harvesters as heavy. However, 6.3% of the participants did a combination of tasks in the 7 d leading up to assessment, combining sugarcane harvesting with tasks related to transporting harvested sugarcane. In this situation, the guidelines specify using the heaviest workload activity to determine the estimated metabolic rate, and that activity was sugarcane harvesting (Occupational Safety and Health Administration 2017).

The study showed that in the cooler months, heat stress exposure exceeded the ACGIH-TLV for heat stress for 72.7% of the participants’ working time and 90.9% in the hotter month. In the hotter month, there was only a very short early morning period (06:00 to 07:00) during which average values were below the ACGIH-TLV. However, this period still exceeded the ACGIH-AL. In the cooler months, the initial 2 h of work exposed them to average levels below the TLV, although on some days the AL was exceeded. Similar results were found by others (Radir *et al.*, 2017; Boonruksa et al. 2020; Pundee et al. 2021; Wichatorn and Chaiklieng 2022). Although the ACGIH guidelines do not provide a specific TLV or AL for the allocation of work-rest cycles with 75% to 100% work time within an hour for heavy workloads, this study calculated the TLV and AL based on the representative metabolic rate of 415 W for heavy workload, for comparative purposes. The American Conference of Governmental Industrial Hygienists (2022) states that their standard screening criteria are not recommended for these conditions, since the physiological strain from such work can be significant, regardless of the WBGT. Instead, the guidelines suggest a more detailed approach, including physiological monitoring and a comprehensive analysis, to ensure the safety and well-being of workers performing heavy or very heavy work. To have a more accurate risk assessment, the Predicted Heat Strain Model, as recommended by ISO 7933 (International Organization for Standardization 2023), could be applied. To reduce heat stress, one possibility is that the sugarcane workers could minimize their work during the peak WBGT hours and consider working more in the morning or evening, when temperatures are cooler. Also helpful would be frequent breaks in shaded areas, having plenty of drinking water available, acclimatizing to heat, and less reliance on being paid by the bundle. The methods that the majority of participants used to try to keep cool during breaks were going into the shade of sugarcane plants or nearby trees and using man-made shade to provide temporary shade in the fields. However, these measures were only used during break times and did not mitigate heat exposure during harvesting.

This study assessed individual participant’s heat stress in terms of effective WBGT by considering the participant’s clothing and the measured WBGT. The WBGT_eff_-1hrTWA was also considered because even short exposures can be dangerous, and the WBGT_eff_-1hrTWA is vital for identifying exposures of only a few hours. However, an exposure of only a few minutes is unlikely to be risky unless the temperature is extreme (Occupational Safety and Health Administration 2009). It is important that clothing as one of the heat stress factors is considered (American Conference of Governmental Industrial Hygienists 2022). However, not many studies apply CAF to estimate WBGT. Approximately 77.7% of the participants in the current study wore 2 or 3 layers of clothing on the upper body. It is important to mention that that some participants in this study wore more than 2 layers of clothes and/or also wore hats or face coverings to help protect themselves from direct sun; however, there are no CAF guidelines for such items. Applying a more detailed approach in estimating thermal characteristics of clothing ensembles and examining how body movement and air penetration affect the clothing’s thermal insulation and water vapor resistance, as recommended by ISO 9920 (International Organization for Standardization 2009), could provide valuable insights for advising on clothing choices to reduce heat stress.

As expected, the heat stress in the hotter season was higher than in the cooler season (Lundgren et al. 2014; Venugopal et al. 2015; Langkulsen and Vichit-Vadakan 2018). This is because all the WBGT components (*Tnwb*, *Tdb*, *Tg*, *Rh*, *Av*, and *e*_*a*_) had higher average results in the hotter compared to the cooler month, leading to an increase in heat stress. The Thai Meteorological Department (2023a) reported that the Thai summer season in 2023 started at the beginning of March and lasted until mid-May, during which the weather was generally hotter than other seasons. However, although the differences in heat stress both for the measured and effective WBGT were statistically significant, it was a fairly small difference. Measurements in this study were taken during the end of the cooler season, with the hot season approaching. The results of heat stress, particularly with effective WBGT, tended to be higher among the participants who were involved exclusively with burnt sugarcane harvesting compared to unburnt sugarcane. One possible reason why participants engaged in burnt sugarcane harvesting showed higher effective WBGT might be due to differences in the clothing worn during each harvesting method. This is because the levels of effective WBGT are affected by CAF. The participants who harvested exclusively burnt sugarcane tended to wear more layers of clothes. Also, the seasonal differences in the use of burnt and unburnt sugarcane were primarily due to farmers’ practices. They are more likely to burn sugarcane at the end of the harvesting season, during the hotter months. Harvesting unburnt sugarcane was most in the cooler months, while in the hotter month, burnt sugarcane increased 2-fold. Sugarcane was typically burnt a day before harvesting. There are 4 reasons for burning: to reduce costs, to ease harvesting when cane is damaged by wind or rain, due to accidental fires, or because of tight timelines before the sugar mills’ order deadline (approximately 23 to 25 March 2023). However, the Thai government promotes harvesting fresh sugarcane to reduce exposure to fine particulate matter (Thailand Environment Institute Foundation 2022). Regarding correlations between parameters of the WBGT and harvesting methods, only *Rh* and *e*_*a*_ showed a significant relationship to the harvesting method, with higher *Rh* and *e*_*a*_ values observed for harvesting burnt sugarcane and for mixed harvesting in 1 d. This correlation is unexpected and at present unexplainable. It might simply be a coincidence, or it could result from unknown confounding factors. Practically speaking, it is possible that on the burnt harvesting or mixed harvesting days, the weather was coincidently cloudy, leading to an elevation in relative humidity and air humidification. It seems that harvesting method is unlikely to have an actual effect on heat stress parameters. The effect would more likely be due to season. However, the reasons for this are unclear. Research on harvesting methods and heat stress remains limited, highlighting a need for further investigation in this area.

Sugarcane workers work in an informal status in Thailand. All of the participants in this study were sugarcane workers who were part of a temporary seasonal workforce from Northeast Thailand, employed specifically for that sugarcane harvesting period. The results of this study contain information that can be helpful for sugarcane workers and other outdoor workers in Thailand as well as, importantly, organizations or government agencies that take responsibility for the safety and well-being of these workers in identifying measures needed to reduce heat stress levels. This finding should encourage policy development related to heat exposure standards for informal agricultural workers. Guidelines for determining work schedules to reduce exposure during the peak heat stress periods should developed, particularly in the hotter months and when harvesting burnt sugarcane. Training programmes to raise the workers awareness on heat stress and its impact is essential.

### Strengths and limitations

One of the strengths of this study is that the assessment of heat stress was done using WBGT instruments placed in the fields rather than using environmental stations or weather stations. Also, as recommended by Brotherhood (2014) this study measured WBGT index in combination with other environmental parameters such as air temperature and humidity. The Thai sugarcane harvesting period spans both the cool and hot seasons, and this study determined heat stress during both these seasons. Another strength is that heat stress measurements were measured throughout the work shift, so this study was able to identify at what specific time of the working day WBGT standards had been exceeded. A final strength is that the study recorded participants’ clothing while also taking into account the ACGIH-CAF in order to compensate in the WBGT results.

A limitation of this study is that no personal monitoring of heat stress was carried out. Another limitation was that the heat stress measurements in the cooler months were done at the end of the cool season (Mid-January to Mid-February). This might not be a good representation of the cool season as a whole (mid-October to mid-February) because the temperatures are a bit lower during December and January. The WBGT measurement times between the 2 seasons were not the same. Most of the measurements in the cooler months started around 07:00 whereas in the hotter month started around 6:00 because there was limited travel access to the sugarcane fields in the cooler months. If anything, this disparity would reduce rather than inflate differences. The ACGIH-CAF for double-layer clothing was used for the participants who wore 3 layers of shirts, and in fact, increased layers might raise the level of heat stress. Field observations revealed no clear work-rest patterns, with variations between workers. The exact work-rest cycle per hour could not be determined due to the lack of official breaks and self-determined rest periods. Therefore, the work-rest cycle was estimated as an average based on reported daily work and rest durations, assuming consistency throughout the day.

## Conclusion

This study found that the sugarcane workers’ WBGT_eff_-1hrTWA exceeded the ACGIH-TLV for the majority of the work shift in both the cooler and hotter month(s). These Thai sugarcane workers are at significant risk for heat stress, which can lead to heat-related health issues. All WBGT measurements (measured *WBGT*, *Tnwb*, *Tdb*, *Tg*, *Av*, and *e*_*a*_) were statistically significantly higher in the hotter month, except for Rh. Harvesting in the hotter month, as well as harvesting burnt sugarcane and harvesting both burnt and unburnt in 1 d, were significantly associated with increased levels of effective WBGT. Further measures for reducing heat stress levels in both seasons are urgently needed. Sugarcane workers in Thailand typically work within a system of informal employment. The Thai government should take steps to ensure the safety and well-being of these informal workers. Further research would be useful to determine CAF for wearing more than 2 layers of clothes because some sugarcane workers wear 3 layers of clothes.

## Supplementary material

Supplementary material is available at *Annals of Work Exposures and Health* online.

wxaf002_suppl_Supplementary_Material

## Data Availability

Data will be made available upon reasonable request.

## Abbreviations

ACGIH: American Conference of Governmental Industrial Hygienists
AL: action limit
Av: air velocity
CAF: clothing adjustment factor
e_a_: absolute water vapor pressure
e_s_: saturation vapor pressure
FRs: field representatives
LR: lead researcher
OSHA: Occupational Safety and Health Administration
Rh: relative humidity
Tdb: dry-bulb temperature
Tg: globe temperature
TLV: threshold limit value
Tnwb: natural wet-bulb temperature
TWA: time weighted average
WBGT: wet bulb globe temperature
WBGT_eff_-1hrTWA: 1-h time weighted average effective WBGT
