# Supplementary material for: High levels of heat stress among sugarcane workers in Thailand
Source: Ann Work Expo Health. 2025 Feb 3;69(4):401–14. doi: 10.1093/annweh/wxaf002 (PMC12018072; doi:10.1093/annweh/wxaf002)
Supplement: wxaf002_suppl_Supplementary_Material [file wxaf002_suppl_supplementary_material.pdf]

Supplementary material

Title: High levels of heat stress among sugarcane workers in Thailand

Authors: Tadpong Tantipanjaporn[1,2*], Andrew Povey[1], Holly A. Shiels[3], Martie van Tongeren[1]

[1]Centre for Occupational and Environmental Health, School of Health Sciences, Faculty of Biology, Medicine and Health, University of Manchester, Ellen Wilkinson Building (Block C), Oxford Road, Manchester, M13 9PL, United Kingdom

[2]Division of Occupational Health and Safety, Faculty of Public Health, Naresuan University, 99 Moo 9, Thapo Sub-district, Muang District, Phitsanulok City, 65000, Thailand

[3]Division of Cardiovascular Sciences, School of Medical Sciences, Faculty of Biology, Medicine and Health, University of Manchester, Core Technology Facility, 46 Grafton Street, Manchester, M13 9NT, United Kingdom

*Corresponding author: Email: tadpong.tantipanjaporn@postgrad.manchester.ac.uk; tadpongt@nu.ac.th

This supplementary material includes two components: the participant questionnaire and Table S1, which presents R^2 values from univariate general linear models.

Page

1. Participant questionnaire 2

2. Table S1 6

Participant questionnaire

Part 1: Demographic Characteristics					
1.1	**Gender** ☐ 1 = Male ☐ 2 = Female ☐ 3 = Other ☐ DA = Prefer not to answer				
1.2	**Age**		Years		Months
1.3	**What is the highest level of education you have completed (education years)?**<br>☐ 1 = No education (0 year)<br>☐ 2 = Primary school (6 years)<br>☐ 3 = Lower middle school (9 years)<br>☐ 4 = Higher middle school (12 years)<br>☐ 5 = Technical school (12 year)<br>☐ 6 = Higher technical school (14 year)<br>☐ 7 = Associates degree college (14 years)<br>☐ 8 = Bachelor's degree (16 years)<br>☐ 9 = More than bachelor's degree (more than 16 years)<br>☐ 10 = Other, please specify …………………<br>☐ DA = Prefer not to answer				

Section 2: Working Conditions	
2.1	**Working Experience**<br>How long have you been working this job (sugarcane harvesters or sugarcane famer)?<br>*(Please specify)* _________ years ________months<br>☐ UN = Do not recall
2.2	**Task in the past 7 days**<br>In the past 7 days, what was your main responsibility in this harvesting stage of sugarcane?<br>☐ 1 = Harvesting ☐ 2 = Other *(please specify)* ____________ ☐ UN = Do not recall
2.3	**Working hours**<br>2.3.1 In the previous 7 days, how many days did you work?<br>*(Please specify)* _________ Days<br>2.3.2 In the previous 7 days, approximately how many hours did you work per day?<br>*(Please specify)* _________ Hours<br>2.3.3 In the previous 7 days, approximately at what time of day did you start working?<br>*(Please specify)* _________<br>2.3.4 In the previous 7 days, approximately at what time of day did you finish your working task?<br>*(Please specify)* _________
2.4	**Taking breaks**<br>2.4.1 In the past 7 days on an average per day, how many times did you take morning breaks?<br>*(Please specify)* _________ times<br>How long were your usually morning breaks?<br>*(Please specify)* _________ minutes<br>Where did you take morning breaks while working? *(One or more answers is fine.)*<br>☐ 1 = Under a shaded tent/cloth ☐ 4 = Under shaded trees

	☐ 2 = Under a shaded car (beside a car) ☐ 5 = Other area *(please specify)* ______________<br>☐ 3 = Under a shaded structure<br>2.4.2 In the past 7 days on an average per day, how long was <u>your lunch break</u>?<br>(*Please specify)* _________ minutes<br>Where did you take lunch break? *(One or more answers is fine.)*<br>☐ 1 = Under a shaded tent/cloth ☐ 4 = Under shaded trees<br>☐ 2 = Under a shaded car (beside a car) ☐ 5 = Other area *(please specify)* ______________<br>☐ 3 = Under a shaded structure<br>2.4.3 In the past 7 days on an average per day, how many times did you take <u>afternoon breaks</u>?<br>(*Please specify)* _________ times<br>How long were your usually afternoon breaks per period?<br>(*Please specify)* _________ minutes<br>Where did you take afternoon breaks? *(One or more answers is fine.)*<br>☐ 1 = Under a shaded tent/cloth ☐ 4 = Under shaded trees<br>☐ 2 = Under a shaded car (beside a car) ☐ 5 = Other area *(please specify)* ______________<br>☐ 3 = Under a shaded structure
2.5	At your current workplace, are any of the following available to help keep you cool during breaks? *(One or more answers is fine.)*<br>☐ 1 = Shade structure<br>☐ 2 = Trees<br>☐ 3 = Fans<br>☐ 4 = Tent<br>☐ 5 = Rest stations such as shack in a field<br>☐ 6 = Building with air conditioning<br>☐ 7 = Other cooling methods not listed here *(please specify)* ______________<br>☐ 8 = There are no cooling methods available at work

Section 3: Clothing Characteristics

3.1 Did you unbutton, unzip, or remove any layer of clothing whenever they felt hot in the past 7 days ?

☐ 1 = Yes ☐ 2 = No

3.2 **Head protection**

Did you wear head protection gear in the past 7 days?

☐ 1 = Yes (Please answer next question) ☐ 2 = No

What type of main head protection gear did you wear in the past 7 days?

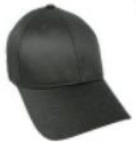

☐ 1 = Cap

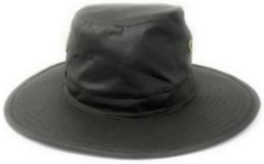

☐ 2 = Wide-brimmed hat

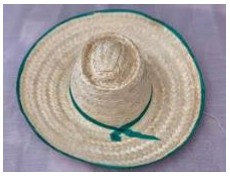

☐ 3 = Wide-brimmed straw hat

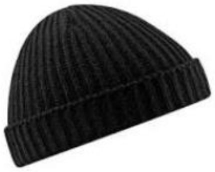

☐ 4 = Beanie

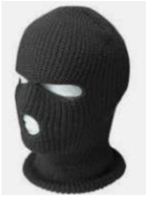

☐ 5 = Balaclava/ski-mask

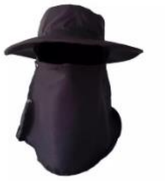

☐ 6 = Shawl sun hat

☐ 7 = Other *(please specify)* ___________________________

3.3 **Shirt**

What type of the main shirt did you wear <u>in the past 7 days</u>? How many layers of shirts did you wear?

(**Note:** *can choose more than one answer*)

☐ 1 = Shirt 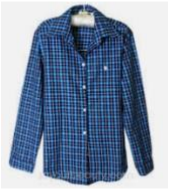 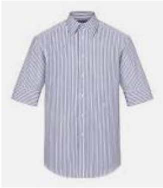	☐ 1.1 = Long-sleeved cotton	Layer (s) _____
	☐ 1.2 = Short-sleeved cotton	Layer (s) _____
	☐ 1.3 = Long-sleeved polyester	Layer (s) _____
	☐ 1.4 = Short-sleeved polyester	Layer (s) _____
	☐ 1.5 = Long-sleeved cotton/polyester blend	Layer (s) _____
	☐ 1.6 = Short-sleeved cotton/polyester blend	Layer (s) _____
	☐ 1.7 = Other *(please specify)* ______________	Layer (s) _____
☐ 2 = T-shirt 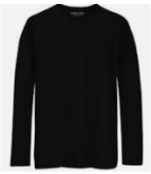 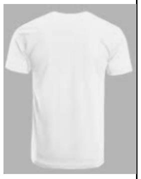	☐ 2.1 = Long-sleeved cotton	Layer (s) _____
	☐ 2.2 = Short-sleeved cotton	Layer (s) _____
	☐ 2.3 = Long-sleeved polyester	Layer (s) _____
	☐ 2.4 = Short-sleeved polyester	Layer (s) _____
	☐ 2.5 = Long-sleeved cotton/polyester blend	Layer (s) _____
	☐ 2.6 = Short-sleeved cotton/polyester blend	Layer (s) _____
	☐ 2.7 = Other *(please specify)* ______________	Layer (s) _____

	☐ 3 = Jacket<br>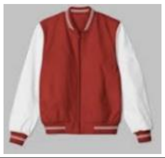	☐ 3.1 = Cotton	Layer (s) _____
		☐ 3.2 = Polyester	Layer (s) _____
		☐ 3.3 = Cotton/polyester blend	Layer (s) _____
		☐ 3.4 = Other *(please specify)* ______________	Layer (s) _____
	☐ 4 = Other *(please specify)* ______________		Layer (s) _____
3.4	**Trousers**<br>What type of the main trousers did you wear <u>in the past 7 days</u>? How many layers of trousers did you wear? (**Note:** *can choose more than one answer*)		
	☐ 1 = Long trousers<br>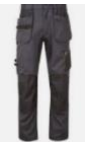 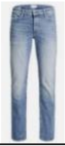	☐ 1.1 = cotton	Layer (s) _____
		☐ 1.2 = polyester	Layer (s) _____
		☐ 1.3 = cotton/polyester blend	Layer (s) _____
		☐ 1.4 = Other *(please specify)* ______________	Layer (s) _____
	☐ 2 = Shorts<br>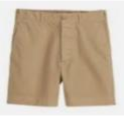	☐ 2.1 = cotton	Layer (s) _____
		☐ 2.2 = polyester	Layer (s) _____
		☐ 2.3 = cotton/polyester blend	Layer (s) _____
		☐ 2.4 = Other *(please specify)* ______________	Layer (s) _____
	☐ 3 = Other *(please specify)* ______________		Layer (s) _____

Table S1. R^2 from univariate general linear models

Explanatory variable	Measured WBGT	Tnwb	Tdb	Tg	Rh	Av	e_a
	**R^2**	**R^2**	**R^2**	**R^2**	**R^2**	**R^2**	**R^2**
Season	0.34	0.32	0.47	0.10	0.08	0.05	0.24
Harvesting method	0.14	0.19	0.16	0.01	0.20	0.01	0.24
Measurement location	0.00	0.00	0.00	0.00	0.00	0.00	0.00

Measured WBGT = Measured wet bulb globe temperature; Tnwb = natural wet-bulb temperature; Tdb = dry-bulb temperature; Tg = globe temperature; Rh = relative humidity; Av = air velocity; e_a = absolute water vapor pressure
n = 50 measurements (2 locations*25 days) for WBGT, Tnwb, Tdb, Tg, and Rh; n = 290 measurements (5-6 times*2 points*25 days) for Av; e_a, calculated from Rh and Tdb, resulted in 50 values.
